# The Influence of the Hyaluronic Acid Gel on the Postoperative Sequelae following Surgical Removal of the Impacted Mandibular Third Molar in Comparison with the A-PRF: A Randomized Controlled Trial

**DOI:** 10.1155/2023/1883460

**Published:** 2023-04-29

**Authors:** Thoulfokar Shokor Al-Saadi, Ahmed Fadhel Al-Quisi

**Affiliations:** Oral and Maxillofacial Surgery Department, College of Dentistry, University of Baghdad, Baghdad, Iraq

## Abstract

One of the most common procedures in oral surgery is the removal of impacted mandibular third molars, often followed by pain, swelling, alveolitis, and trismus. *Purpose*. To compare the outcomes of the intrasocket application of 1% hyaluronic acid oral gel (HA) and advanced platelet-rich fibrin (A-PRF) on the expected postoperative complications, pain, swelling, and trismus follow the surgical extraction of the impacted mandibular third molar. *Material and Methods*. A randomized controlled trial was conducted at the Oral and Maxillofacial Surgery Unit, Dental Teaching Hospital. Healthy patients who required surgical removal of the impacted mandibular third molar were divided randomly into three groups. The extraction site of the group (A) patients remained without the addition of any material, just suturing of the wound with simple interrupted sutures, while in group (B) patients, the extraction site was filled with 1 cc of 1% hyaluronic acid gel (periokin®), and in group C patients, the extraction site was filled with A-PRF. *Results*. Sixty-six eligible patients participated in this study; both hyaluronic acid gel 1% (periokin®) and advanced platelet-rich fibrin showed a significant reduction in pain, swelling, and trismus on the 1st, third, and seventh postoperative days when compared to the control group, while the comparison between HA and A-PRF showed no significant differences except for the pain on the third postoperative day. There was a significant pain decrease in the A-PRF group than HA group. *Conclusion*. Intrasocket application of 1% hyaluronic acid gel (periokin®) or advanced platelet-rich fibrin can be an effective primary way to significantly reduce postoperative pain, trismus, and edema compared to the control group following mandibular third molar surgery.

## 1. Introduction

Pain, edema, and trismus are the most common postoperative sequels seen in individuals who underwent oral surgical procedures. Other clinical signs, including facial or neck hematomas, persistent alveolus bleeding, pyrexia, and dry socket symptoms, may be observed. These symptoms are prevalent after mandibular third molar surgical removal, considered one of the most challenging and time-consuming oral surgical procedures [1].

The severity of these postoperative sequels is directly related to the depth and space available for removal of the impacted mandibular third molar, the angulation of the tooth, root spacing, size of the bone septum, presence or absence of a dilated tooth follicle, periodontal space, bone density, and the relation to the inferior alveolar nerve [2]. Delays in recovery and an increased chance of developing chronic pain have been linked to inadequate care for early postoperative pain [3]. These morbidities remained a significant issue for patients and surgeons; as a result, decreasing these problems may significantly improve patient outcomes [4].

Nowadays, platelet-rich fibrin (PRF), also known as an autologous fibrin matrix, is a second-generation platelet concentrate that is considered a valuable factor in reducing discomfort associated with the postodontectomy healing phase; these autologous materials are derived from the patient's blood constituents to regulate inflammation and aid the healing process [5–7]. It does not induce allergies and poses no danger of cross-infection. It comprises a three-dimensional fibrin matrix rich in platelets and leukocytes. It contains cytokines, stem cells, and growth factors, forming a biodegradable scaffold that promotes microvascularization and facilitates epithelial cell migration to its surface [8].

Furthermore, platelet-rich fibrin concentrates can release growth factors for 1 to 4 weeks, ensuring a longer duration of healing stimulation than platelet-rich plasma, which releases all growth factors at the time of administration [6].

Advanced platelet-rich fibrin (A-PRF) can be obtained by lowering the centrifugation speed, which leads to more efficient uptake of cells and cytokines during centrifugation through developing a persistent fibrin net [9].

On the other hand, several studies showed that hyaluronic acid (HA) is a good option for accelerating wound healing by promoting granulation tissue development, preventing destructive inflammation during the healing phase, and performing re-epithelialization and angiogenesis [10]. Hyaluronic acid is a naturally derived polymer biomaterial. It is a major extracellular matrix (ECM) component in nearly every mammalian tissue and fluid [11].

It mediates chemotaxis, proliferation, and progressive differentiation of mesenchymal cells. It, therefore, plays a vital role in tissue regeneration and repair [12].

This study compares the outcomes of 1% hyaluronic acid oral gel and advanced platelet-rich fibrin on the expected postoperative sequels (pain, swelling, and trismus) following the surgical extraction of the impacted mandibular third molar.

## 2. Materials and Methods

A double-blind, randomized controlled trial was conducted in the Oral and Maxillofacial Surgery Unit, Dental Teaching Hospital, according to the ethical principles and in compliance with the Declaration of Helsinki and its later amendments.

This study had been ethically approved by The Research Ethics Committee of the College of Dentistry, University of Baghdad, with a reference number (393) on the date 27-12-2021. This trial had been registered at the Thai clinical trial registry with a registration number (TCTR 20220602008).

A thorough clinical and radiological examination by an independent maxillofacial surgeon was performed on all patients in this study, and only healthy, none smoker patients with horizontal and mesioangular impacted lower third molar teeth (level A or B, class I or II) according to winter classification and the degree of impaction based on the Pell & Gregory category classification were included in this study.

The sample size of this study was calculated by using G-power software. Block randomization was achieved with Microsoft Excel 2013 to ensure all groups had the same number of patients. Moreover, an independent Maxillofacial Surgeon made subsequent evaluations of the pain, swelling, and trismus during the three visits (first, third, and seventh days following the surgery).

The eligible patients were randomly divided into three groups. Group A patients (control group) were managed by surgical extraction of the impacted lower third molar tooth without placing material inside the extraction socket. Group B patients (study group) were managed by applying 1 cc of 1% hyaluronic acid gel (periokin® Spain) combined with gel foam in a sterilized amalgam jar to avoid slippage of the material out of the surgical site. Group C patients (study group) were managed by applying A-PRF into the extraction socket after surgical removal of the impacted mandibular third molar.

**Figure 1:**
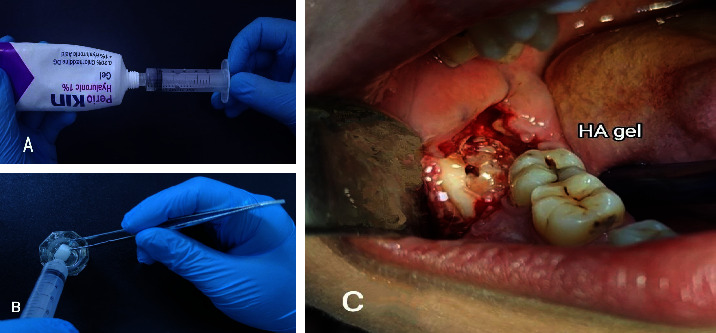
Hyaluronic acid gel preparation and mixing with gel foam: (a) 1 cc of hyaluronic acid gel (periokin®) was drawn by using a plastic hypodermic syringe, (b) mixing gel foam (Spongostan®, Denmark) with the hyaluronic acid gel in a sterilized amalgam jar, and (c) application of the mixture into the surgical site.

**Figure 2:**
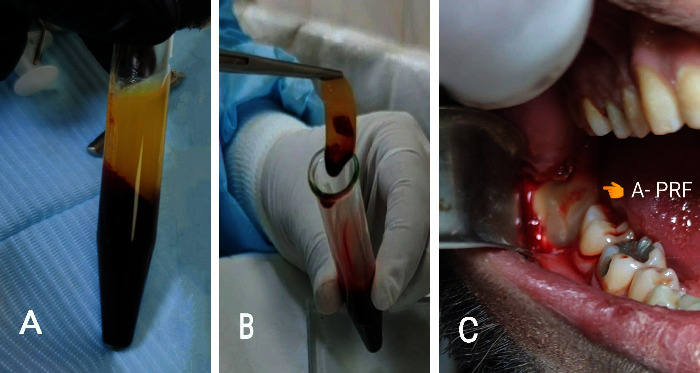
Clinical case for the preparation of A-PRF: (a) after centrifugation of the blood, the tube shows two layers (A-PRF at the top and RBC at the bottom), (b) picking up A-PRF from the tube, and (c) A-PRF application into the socket.

Before the surgical procedure, all patients were instructed to rinse their mouths with 0.12% chlorhexidine mouthwash for 30–60 seconds. The patients were operated under the local anesthesia obtained by block injection of 1.8 ml of 2% lidocaine hydrochloride with epinephrine 1 : 80,000 (Huon's Co., Ltd., Korea) into the inferior alveolar nerve with infiltration anesthesia into the long buccal and lingual nerves. The same operator performed all the surgical procedures, where a full mucoperiosteal flap was raised after a triangle incision with a no. 15 scalpel blade. The osteotomy was done with a round bur mounted on a W&H surgical low-speed handpiece. Tooth extraction was performed using an elevator following adequate bone osteotomy and tooth sectioning. Following tooth removal, the socket was irrigated with chlorhexidine 0.2%. Then, the wound was sutured with simple interrupted sutures for the group A patients. The extraction site of the group B patients was filled with one cc of 1% hyaluronic acid gel (periokin®) mixed with gel foam, as shown in Figure 1. In group C patients, A-PRF was prepared using 10 ml of the patient's venous blood drawn from the median cubital vein and inserted immediately into dry, anticoagulant-free, glass-coated plastic tubes as shown in Figure 2. The centrifuging time was 14 minutes, and the speed was 1500 rpm [13]. A-PRF is the yellowish layer formed at the top of the centrifuged tube that is dissected by scissors from the red corpuscle base at the bottom and applied to the surgical site before suturing.

The preoperative assessment includes measurement of the maximum mouth opening, and the postoperative assessment includes the pain, trismus, and swelling. The pain was measured using a visual analog scale (VAS) from a zero to 10 score, where zero represents the lowest pain value, and ten is the highest. The maximum interincisal opening was used to assess the trismus.

Facial swelling was subjectively assessed by criteria mentioned by Sulieman [14] as follows: grade 0 represents no swelling, grade 1 represents edema of alveolar mucosa buccally or lingually (intraorally), grade 2 represents edema of alveolar mucosa buccally or lingually and involves the cheek (extra-orally) to the body of the mandible, and grade 3 represents edema of alveolar mucosa buccally or lingually and involves the cheek (extra-orally) below the body of the mandible.

Follow-up assessment was done on the first, third, and seventh postoperative days. The statistical analysis was performed using Statistical Package for Social Science (SPSS version 21, Chicago In press, Illinois, USA). A Shapiro-Wilk test was performed to evaluate the normality of the data distribution. The chi-square test assessed the distribution association between two qualitative variables when the expected cell counts less than 5 is <20%, as in the demographic data. Dunn-Bonferroni method was performed to test whether multiple pairs of samples are significantly different. The probability value was considered significant when it was less than 0.05.

## 3. Results

**Figure 3:**
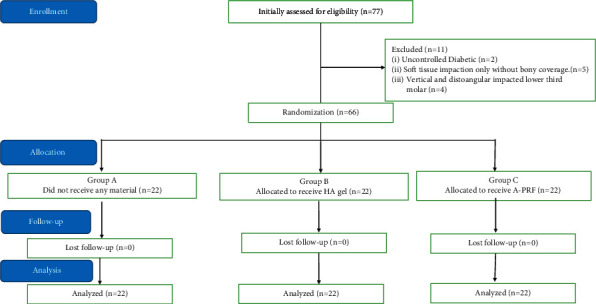
The flowchart illustrates the study's basic steps.

Seventy-seven individuals with 77 impacted mandibular third molars took part in this study; 11 patients were excluded because they did not meet the inclusion criteria, and 66 patients were randomly divided into three groups (3), as shown in Figure 3.

The result of the current study demonstrated that the patient's ages ranged from 18–29 years old, with 37 females and 29 males. There were no significant differences between age, gender, side of impaction, and the impaction angle among groups, as shown in Table 1.

The statistical analyses showed a significant difference among all groups on all postoperative days except between the HA and A-PRF groups on the first and seventh postoperative days, as shown in Table 2. The decrease in pain during the postoperative days was more in the A-PRF group, followed by the HA group, and the slightest decrease was in the control group.

Regarding the swelling, statistical analyses showed significant differences among groups on all postoperative days except between HA and A-PRF groups, as shown in Table 3. The grade of swelling was higher in the control group on all postoperative days than in the HA and A-PRF groups.

Regarding the trismus, Multiple pairwise comparisons (Tukey's HSD method) showed highly significant differences among all groups on all postoperative days except between the HA and A-PRF groups, as shown in Table 4.

## 4. Discussion

Pain, swelling, and trismus are the most common postoperative inflammatory reactions after surgically removing the impacted mandibular third molar [15].

Regarding pain, the visual analog scale (VAS) was employed in this study to measure the severity of pain felt after surgical removal of the impacted mandibular third molar because it is reproducible, straightforward, and easy for the patient to understand.

This study showed that pain significantly decreased in both HA and A-PRF groups compared to the control group. These results agreed with several previous studies demonstrating the effectiveness of HA gel or A-PRF in reducing postoperative pain [1, 16, 17].

However, when comparing the results of HA and A-PRF groups regarding pain reduction, a statistically significant decrease on the third postoperative day is only in favor of the A-PRF group; this result may be explained by the fact that A-PRF aids in the acceleration of healing and angiogenesis and minimizes the danger of inflammation in the region of application [18–20]. Since the severity of the postoperative pain resulting from minor oral surgeries usually fades in days following the surgery [21], it might explain the disappearance of A-PRF preference over hyaluronic acid on the seventh postoperative day.

Deliverska and Petkova [22] proposed that postsurgical edema might be caused by the tissues' reaction to manipulation and trauma during surgery. Besides that, the length of the incision and the duration of the surgery can affect the swelling form; therefore, smaller incisions result in substantially less postoperative edema [23–25].

In this study, the swelling began immediately after surgical removal of the impacted mandibular third molar and gradually increased to reach its maximum degree on the first and second postoperative days, then gradually subsided on the third or fourth postoperative days, and this agreed with many previous studies [26–28]. This study showed a statistically significant reduction in facial swelling in both the HA and A-PRF groups compared to the control group, which agrees with previous studies [17, 18, 20], with no statistically significant difference between HA and A-PRF groups in all postoperative days.

The antiedematous properties of HA could be attributed to its osmotic buffering capabilities in addition to delaying leukocyte migration via adhering to its receptor CD44 [29, 30], and the antiedematous properties of A-PRF could be attributed to its ability in minimizing the risk of inflammation in the surgical site [1].

On the other hand, the typical and expected result of third molar surgery is trismus, which is measured by comparing the pre- and postoperative maximum interincisal distance [22]. Balakrishnan et al. found that pain is the leading cause of trismus following the extraction of impacted mandibular third molars [31]. The excellent performance of both A-PRF and HA regarding pain reduction may explain their statistically significant reduction in the trismus on the first, third, and seventh postoperative days compared to the control group.

However, this result disagreed with Yilmaz et al. [32, 33] studies, which may be attributed to the small sample size of these studies. Furthermore, this study showed no significant difference between the HA and A-PRF groups regarding the trismus on postoperative days.

In conclusion, 1% HA gel (periokin®) or advanced platelet-rich fibrin can significantly reduce postoperative pain, trismus, and edema after mandibular third molar surgery. However, the easy use reduced HA preparation time, and the patient's disturbing blood drawing makes HA superior to A-PRF.

## Figures and Tables

**Table 1:** A detailed demographic feature regarding different parameters for all groups.

Vars		Groups						Chi-square	*P* value
		Control Mean = 23.3 SD = ±3.5		H.A Mean = 23.05 SD = ±2.9		A-PRF Mean = 22.9 SD = ±2.9			
		*N*	%	*N*	%	*N*	%		
Age (years)	18–23	11	16.66	12	18.18	13	19.69	0.121	0.941
	24–29	11	16.66	10	15.15	9	13.65		

Gender	Female	10	15.15	14	21.21	13	19.69	1.599	0.449
	Male	12	18.18	8	12.12	9	13.65		

Side of impaction	Left	11	50.00	10	45.45	10	45.45	0.122	0.941
	Right	11	50.00	12	54.55	12	54.55		

Winter classification	Horizontal	13	59.09	10	45.45	12	54.55	0.852	0.653
	Mesioangular	9	40.91	12	54.55	10	45.45		

Pell and Gregory classification	CL1 level A	4	18.18	5	22.73	5	22.73	0.756	0.993
	CL1 level B	4	18.18	5	22.73	5	22.73		
	CL2 level B	7	31.82	7	31.82	7	31.82		
	CL2 level A	7	31.82	5	22.73	5	22.73		

**Table 2:** Multiple pairwise comparisons of pain among the groups by using the Dunn-Bonferroni method.

Period	Groups		*P* value
1-day postoperatively	Control	HA	0.001^*∗∗*^
	Control	A-PRF	0.001^*∗∗*^
	HA	A-PRF	0.277

3-day postoperatively	Control	HA	0.001^*∗∗*^
	Control	A-PRF	0.001^*∗∗*^
	HA	A-PRF	0.031^*∗*^

7-day postoperatively	Control	HA	0.036^*∗*^
	Control	A-PRF	0.008^*∗∗*^
	HA	A-PRF	1.00

^*∗*^Significant, ^*∗∗*^highly significant.

**Table 3:** Multiple pairwise comparisons of swelling among the groups by using the Dunn-Bonferroni method.

Period	Groups		*P* value
1-day postoperatively	Control	HA	0.001^*∗∗*^
	Control	A-PRF	0.001^*∗∗*^
	HA	A-PRF	1.00

3-day postoperatively	Control	HA	0.001^*∗∗*^
	Control	A-PRF	0.001^*∗∗*^
	HA	A-PRF	1.00

7-day postoperatively	Control	HA	0.031^*∗*^
	Control	A-PRF	0.031^*∗*^
	HA	A-PRF	1.00

^*∗*^Significant, ^*∗∗*^highly significant.

**Table 4:** Multiple pairwise comparisons of trismus among groups by using (Tukey's HSD).

Period	Groups		*P* value
1-day postoperative	Control	HA	0.001^*∗∗*^
	Control	A-PRF	0.001^*∗∗*^
	HA	A-PRF	1.00

3-day postoperative	Control	HA	0.001^*∗∗*^
	Control	A-PRF	0.001^*∗∗*^
	HA	A-PRF	1.00

7-day postoperative	Control	HA	0.001^*∗∗*^
	Control	A-PRF	0.001^*∗∗*^
	HA	A-PRF	1.00

^*∗∗*^Highly significant.

## Data Availability

The data used to support the findings of this study are available from the corresponding author upon request.

## References

[1] Starzyńska A., Kaczoruk-Wieremczuk M., Lopez M. A., Passarelli P. C., Adamska P. (2021). The growth factors in advanced platelet-rich fibrin (A-PRF) reduce postoperative complications after mandibular third molar odontectomy. *International Journal of Environmental Research and Public Health*.

[2] Barone S., Antonelli A., Averta F. (2021). Does mandibular gonial angle influence the eruption pattern of the lower third molar? A three-dimensional study. *Journal of Clinical Medicine*.

[3] Shabat M. A., Bede S. Y. (2021). Effect of the local application of bupivacaine in early pain control following impacted mandibular third molar surgery: a randomized controlled study. *Dental and Medical Problems*.

[4] Jamil F., Al-Quisi A., Asmael H. (2022). The effectiveness of limited flap design in surgical removal of fully impacted lower third molars: a comparative clinical study. *Journal of Stomatology*.

[5] Abbas A. M., Bede S. Y., Alnumay S. H. (2019). Evaluation of the effectiveness of using platelet rich fibrin (PRF) as a sole grafting material and membrane in augmentation of dehiscence and fenestration defects encountered during dental implant surgery. *Journal of Baghdad College of Dentistry*.

[6] Brancaccio Y., Antonelli A., Barone S., Bennardo F., Fortunato L., Giudice A. (2021). Evaluation of local hemostatic efficacy after dental extractions in patients taking antiplatelet drugs: a randomized clinical trial. *Clinical Oral Investigations*.

[7] Al-Mahdi A. H., Abdulrahman M. S., Al-Jumaily H. A. H. (2021). Evaluation of the effectiveness of using platelet rich fibrin (PRF) with bone graft in the reconstruction of alveolar cleft, a prospective study. *Journal of Craniofacial Surgery*.

[8] Upadhayaya V., Arora A., Goyal A. (2017). Bioactive platelet aggregates: prp, prgf, prf, cgf and sticky bone. *IOSR Journal of Dental and Medical Science*.

[9] Kareem A. S. A., Al Hussaini A. H. (2019). Effect of platelet rich-fibrin on alveolar osteitis incidence following surgical removal of impacted mandibular third molars: a comparative study. *Journal of Baghdad College of Dentistry*.

[10] Rohde L. E., Clausell N., Ribeiro J. P. (2005). Health outcomes in decompensated congestive heart failure: a comparison of tertiary hospitals in Brazil and United States. *International Journal of Cardiology*.

[11] Radhi I. H., Al-Ghaban N. M. (2015). Evaluation the effect of hyaluronic acid on bone healing process in rabbits (Immunohistochemical study for TGF-*β*). *Journal of baghdad college of dentistry*.

[12] Mohammad M. H., Al-Ghaban N. M. (2018). Histological and histomorphometric studies of the effects of hyaluronic acid on osseointegration of titanium implant in rabbits. *Journal of baghdad college of dentistry*.

[13] Shah R., Thomas R., Thomas R., Mehta D. S. (2017). An update on the protocols and biologic actions of platelet rich fibrin in Dentistry. *The European Journal of Prosthodontics and Restorative Dentistry*.

[14] Sulieman M. S. (2004). Clinical evaluation of the effect of four flap designs on the post–operative sequel (pain, swelling and trismus) following lower third molar surgery. *Al-Rafidain Dental Journal*.

[15] Srivastava N., Shetty A., Kumar P., Rishi D., Bagga V., Kale S. G. (2021). Comparison of preemptive effect of dexamethasone and methylprednisolone after third molar surgery: a split-mouth randomized triple-blind clinical trial. *Journal of Maxillofacial and Oral Surgery*.

[16] Muñoz-Cámara D., Pardo-Zamora G., Camacho-Alonso F. (2021). Postoperative effects of intra-alveolar application of 0.2% chlorhexidine or 1% hyaluronic acid bioadhesive gels after mandibular third molar extraction: a double-blind randomized controlled clinical trial. *Clinical Oral Investigations*.

[17] Nariman S. K., Al-kamali R. K. (2021). Clinical efficacy of hyaluronic acid in post-extraction sockets of impacted mandibular third molar. *Erbil Dental Journal (EDJ)*.

[18] Kargarpour Z., Nasirzade J., Panahipour L., Mitulović G., Miron R. J., Gruber R. (2021). Platelet-rich fibrin increases BMP2 expression in oral fibroblasts via activation of TGF-*β* signaling. *International Journal of Molecular Sciences*.

[19] Kargarpour Z., Nasirzade J., Panahipour L., Miron R. J., Gruber R. (2020). Relative centrifugal force (RCF; G-force) affects the distribution of TGF-*β* in PRF membranes produced using horizontal centrifugation. *International Journal of Molecular Sciences*.

[20] Mazor Z., Horowitz R. A., Del Corso M., Prasad H. S., Rohrer M. D., Dohan Ehrenfest D. M. (2009). Sinus floor augmentation with simultaneous implant placement using Choukroun’s platelet‐rich fibrin as the sole grafting material: a radiologic and histologic study at 6 months. *Journal of Periodontology*.

[21] Sanari A. A., Alsolami B. A., Abdel-Alim H. M., Al-Ghamdi M. Y., Meisha D. E. (2020). Effect of smoking on patient-reported postoperative complications following minor oral surgical procedures. *The Saudi Dental Journal*.

[22] Deliverska E. G., Petkova M. (2016). Complications after extraction of impacted third molars-literature review. *Journal of IMAB - Annual Proceeding (Scientific Papers)*.

[23] Shevel E., Koepp W. G., Bütow K. W. (2001 May). A subjective assessment of pain and swelling following the surgical removal of impacted third molar teeth using different surgical techniques. *South African Dental Journal*.

[24] Syed K. B., AlQahtani F. H. K., Mohammad A. H. A., Abdullah I. M., Qahtani H. S. H., Hameed M. S. (2017). Assessment of pain, swelling and trismus following impacted third molar surgery using injection dexamethasone submucosally: a prospective, randomized, crossover clinical study. *Journal of International Oral Health*.

[25] Kim K., Brar P., Jakubowski J., Kaltman S., Lopez E. (2009). The use of corticosteroids and nonsteroidal antiinflammatory medication for the management of pain and inflammation after third molar surgery: a review of the literature. *Oral Surgery, Oral Medicine, Oral Pathology, Oral Radiology and Endodontics*.

[26] Singh N., Kumar M., Singh S., Srivastava A. S. R. (2020). Primary closure versus secondary closure after third molar surgery: a comparative evaluation of postooperative sequelae. *International Journal of Contemporary Medicine, Surgery and Radiology*.

[27] Kuma N. (2021). DRY socket etiopathogenesis, management and prevention: a brief systematic review of literature. *European Journal of Molecular and Clinical Medicine*.

[28] Zahid T. M., Nadershah M. (2019). Effect of advanced platelet-rich fibrin on wound healing after third molar extraction: a split-mouth randomized double-blind study. *The Journal of Contemporary Dental Practice*.

[29] Cooper C. A., Brown K. K., Meletis C. D., Zabriskie N. (2008). Inflammation and hyaluronic acid. *Alternative and Complementary Therapies*.

[30] Longinotti C. (2014). The use of hyaluronic acid based dressings to treat burns: a review. *Burns and trauma*.

[31] Balakrishnan G., Narendar R., Kavin T., Venkataraman S., Gokulanathan S. (2017). Incidence of trismus in transalveolar extraction of lower third molar. *Journal of Pharmacy and BioAllied Sciences*.

[32] Yilmaz N., Demirtas N., Kazancioglu H., Bayer S., Acar A., Mihmanli A. (2017). The efficacy of hyaluronic acid in postextraction sockets of impacted third molars: a pilot study. *Nigerian Journal of Clinical Practice*.

[33] Torul D., Omezli M., Kahveci K. (2020). Evaluation of the effects of concentrated growth factors or advanced platelet rich-fibrin on postoperative pain, edema, and trismus following lower third molar removal: a randomized controlled clinical trial. *Journal of stomatology, oral and maxillofacial surgery*.

